# Performance Evaluation and Requirements Assessment for Gravity Gradient Referenced Navigation 

**DOI:** 10.3390/s150716833

**Published:** 2015-07-13

**Authors:** Jisun Lee, Jay Hyoun Kwon, Myeongjong Yu

**Affiliations:** 1 Department of Geoinformatics, University of Seoul, Seoul 130-743, Korea; E-Mail: leejs@uos.ac.kr; 2 Department of Civil, Environmental and Geodetic Engineering, Ohio State University, Columbus, OH 43210, USA; 3 Agency for Defense Development, Daejeon 151-742, Korea; E-Mail: mjyu@add.re.kr

**Keywords:** gravity gradient, GGRN, EKF, requirements analysis

## Abstract

In this study, simulation tests for gravity gradient referenced navigation (GGRN) are conducted to verify the effects of various factors such as database (DB) and sensor errors, flight altitude, DB resolution, initial errors, and measurement update rates on the navigation performance. Based on the simulation results, requirements for GGRN are established for position determination with certain target accuracies. It is found that DB and sensor errors and flight altitude have strong effects on the navigation performance. In particular, a DB and sensor with accuracies of 0.1 E and 0.01 E, respectively, are required to determine the position more accurately than or at a level similar to the navigation performance of terrain referenced navigation (TRN). In most cases, the horizontal position error of GGRN is less than 100 m. However, the navigation performance of GGRN is similar to or worse than that of a pure inertial navigation system when the DB and sensor errors are 3 E or 5 E each and the flight altitude is 3000 m. Considering that the accuracy of currently available gradiometers is about 3 E or 5 E, GGRN does not show much advantage over TRN at present. However, GGRN is expected to exhibit much better performance in the near future when accurate DBs and gravity gradiometer are available.

## 1. Introduction

As a typical dead-reckoning system, an inertial navigation system (INS) determines the current position by integrating measurements from accelerometers and gyroscopes with respect to a prior position. The advantage of the INS is that it calculates the position and attitude of a vehicle without using other supplemental sensors. However, position and attitude errors are cumulative with respect to time owing to sensor errors, initial position and attitude errors, and the dynamics of vehicles [1,2]. Satellite navigation systems known as global navigation satellite systems (GNSSs) provide high-precision positioning on the basis of radio signals transmitted from satellites. Unlike the INS, positioning errors from a GNSS are not cumulative with respect to time, so the latter system has long-term stability. The Global Positioning System (GPS), GLObal NAvigation Satellite System (GLONASS), and Galileo are notable examples of a GNSS. However, such systems are vulnerable to hostile environments such as signal jamming because the strength of the signal from the satellite is weak. Solar storms and intentional jamming are examples of events posing a risk to a GNSS. Therefore, a combined navigation system termed GNSS/INS has been developed to overcome these drawbacks of both the individual navigation systems [3,4,5,6]. However, this system is also unable to determine a position with high accuracy in a non-GNSS environment. In such situations, an alternative method for determining the position precisely and compensating for INS errors is required. 

One of the methods to compensate for INS errors is database referenced navigation (DBRN), which uses geophysical information such as gravity, magnetic, and terrain data for navigation. The most popular example of a DBRN system is the terrain referenced navigation (TRN) system. The positioning accuracy of TRN is known to be on the order of several tens of meters, and various TRNs, such as terrain contour matching (TERCOM) and Sandia inertial terrain-aided navigation (SITAN), have already been utilized for the positioning of airplanes, submarines, and missiles [7,8,9]. However, gravity and magnetic field data have not been employed widely, owing to difficulties in developing suitable sensors and achieving their high performance in the survey environment. 

The recent development of the precise gravity gradiometry instrument (GGI) has revived focus on gravity-based navigation, and studies on the mathematical model and feasibility analysis for this navigation have been reported [10,11,12,13]. In previous studies, global geopotential models such as EGM96 and EGM08 were used to construct the gravity gradient DB. The DB constructed from global models would not properly represent the local signatures of the gravity field, so a DB with a higher resolution and accuracy is necessary to increase the reliability of positioning based on the gravity gradient. In addition, in previous studies, the accuracy of the GGI was assumed to be 1 E (E is the unit of the gravity gradient, Eotvos; $1\text{ E}=1\times 10^{-9}/{\text{s}}^{2}$) or higher because those studies focused on the feasibility of GGRN as a future application. However, it is necessary to investigate the possibility of applying the currently available commercial gradiometer having an accuracy of about 3 E or 5 E for navigation. In other words, it would be beneficial to investigate the limitations of current technology and establish the minimum requirements for factors such as the flight altitude, DB resolution and accuracy, and sensor accuracy for GGRN. 

In this study, simulation tests are conducted for evaluating the performance of GGRN under the influence of various factors such as DB and sensor errors, flight altitude, DB resolution, initial errors (position, velocity, and attitude), and measurement update rates. For a more reliable analysis of GGRN, a high-resolution gravity gradient model constructed on the basis of real gravity data is used in the tests. The navigation results from GGRN are compared with those from TRN to analyze the advantages, disadvantages, and limitations of the former. Finally, some conditions for obtaining navigation results with some target accuracies in GGRN are established on the basis of the simulation results. 

## 2. GGRN

The key elements in DBRN are the sensor, DB, and the estimation strategy. In this study, a GGI with a certain accuracy is the main sensor for observing the gravity gradients. A gravity gradient DB is constructed on the basis of real gravity data, and an extended Kalman filter (EKF) for GGRN is developed. It should be mentioned that the gravity gradient DB is not constructed from the real gravity gradient measurement. Instead, the land gravity data and shuttle radar topography mission (SRTM) data are used to simulate a high-resolution gravity anomaly using a topography-correlated model that is adjusted to an existing gravity data [14]. Then, the gravity gradient is calculated by taking second-order derivatives of the disturbing potential that is calculated from the gravity anomaly. Since the gravity gradient is calculated on the basis of the simulated gravity anomaly, it is necessary to verify the consistency and reliability of the simulated gravity anomaly with respect to the real gravity. The statistics of the difference between the real and the simulated gravity anomaly show the mean of 0.5 mGal ($1\text{ mGal}=1\times 10^{-5}\text{ m}/{\text{s}}^{2})$ and the standard deviation of 4.92 mGal, respectively. Considering the statistics, it is supposed that the simulated gravity anomaly reflect the real gravity signal well. This means the simulated gravity gradient is also consistent and reliable. Detailed procedures and results of the gravity gradient modeling can be found in [15]. In the GGRN process, the INS, GGI, and the gravity gradient DB are the key sensors and data sources loaded into a moving vehicle. The GGI is assumed to be assembled on a stabilized platform to minimize the effects of vibrations on measurements. Furthermore, a full-tensor gradiometer (FTG) that senses nine elements is employed in this study. A barometric altimeter and a compass sensor are included as supplemental sensors to compensate for height and yaw errors in the INS. Considering these sensors and data, the GGRN system is constructed using the EKF. 

**Figure 1:**
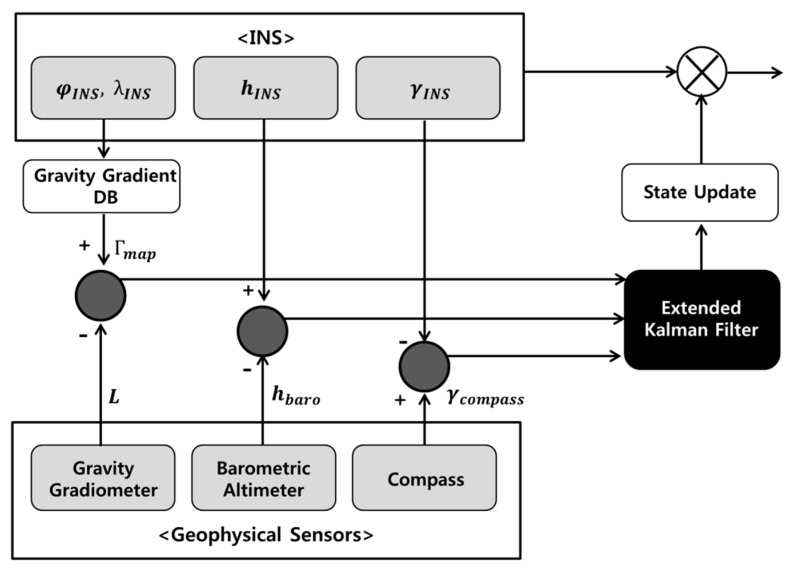
Diagram of gravity gradient referenced navigation.

Figure 1 shows the principles of GGRN. First, the INS computes the current position, velocity, and attitude by integrating the measurement from the inertial measurement unit (IMU). The geophysical sensors, including the GGI, altimeter, and compass, are assumed to acquire gravity gradient, height, and yaw information for the vehicle at every epoch. When the geophysical measurements are obtained, the gravity gradient values corresponding to the INS-indicated position are extracted from the gravity gradient DB. Then, the difference between the gravity gradient obtained from the GGI and that obtained from the DB is used as the measurement in the EKF. Additionally, the differences between the observations from the altimeter and the height from the INS and those between the observations from the compass and the yaw from the INS are used as measurements in the EKF. Using these processes, the EKF estimates 15-state vectors, composed of the position (φ, λ, h), velocity (${\text{v}}_{\text{N}},{\text{ v}}_{\text{E}},{\text{ v}}_{\text{D}})$, attitude (α, β, γ), and accelerometer and gyroscope biases (${\text{b}}_{\text{a},\text{N}},{\text{ b}}_{\text{a},\text{E}},{\text{ b}}_{\text{a},\text{D}},{\text{ b}}_{\text{g},\text{N}},{\text{ b}}_{\text{g},\text{E}},{\text{ b}}_{\text{g},\text{D}}$). In Figure 1, φ, λ, h denote the latitude, longitude, and height, respectively; γ denotes the bearing; and Γ and L denote the gravity gradients from the DB and gradiometer, respectively. The gravity gradient is the spatial rate of change of gravity vector and form a second-order tensor with nine components. In the local North East Down navigation frame, it is given as a symmetric tensor as Equation (1).

(1) $$\text{Γ}=\left(\begin{array}{ccc} \Gamma_{NN} & \Gamma_{NE} & \Gamma_{ND}\\ \Gamma_{NE} & \Gamma_{EE} & \Gamma_{ED}\\ \Gamma_{ND} & \Gamma_{ED} & \Gamma_{DD} \end{array}\right)$$

In GGRN, both Γ and L are composed of five independent components ($\Gamma_{NN}$, $\Gamma_{NE}$, $\Gamma_{ND}$, $\Gamma_{EE}$, $\Gamma_{ED}$) of the gravity gradient tensor, and $\Gamma_{DD}$, which shows the largest value. 

Equation (2) is the observation equation of GGRN. As mentioned earlier, the measurement of the gravity gradient is calculated using the difference between the gravity gradient obtained from the gradiometer at the true position and that obtained from the DB at the INS-indicated position. Because this difference is nonlinear with respect to position, the difference must be linearized before application of the EKF. In addition, both the user clock error and the error rotation vector must be considered because the gradients are assumed to be measured from a stabilized platform. Detailed procedures for deriving the equation for the measurement of the gravity gradient are presented in [10]. The difference between observations from the supplemental sensors (barometric altimeter and compass) and from the INS is calculated and used as measurements to compensate the height and yaw errors from the INS. In this study, it is supposed that the vehicle contains a full-tensor-type gradiometer and six different gravity gradient maps. Therefore, a total of eight measurements are included in each epoch: six for the gravity gradient and two for the height and yaw.

(2) $$z_{k}=H_{k}x_{k}+v_{k},v_{k}\sim N\left(0,R\right)$$

where $z_{k}=[\begin{array}{c} {\delta \Gamma^{n}-\left[\frac{\partial L_{\Psi }^{n}}{\partial \Psi^{n}}\right]\left[\frac{\partial \Psi_{in}^{n}}{\partial cb_{u}}\right]c\delta b_{u}}_{6\times 1}\\ h_{INS}-h_{baro}\\ \gamma_{compass}-\gamma_{INS} \end{array}]$, $H_{k}=\left[\begin{array}{ccccc} {\frac{\partial \Gamma_{\mathrm{map}}}{\partial \varphi }}_{\mathrm{6\times 1}} & {\frac{\partial \Gamma_{\mathrm{map}}}{\partial \lambda }}_{\mathrm{6\times 1}} & 0 & 0_{\mathrm{6\times 12}}\\ 0 & 0 & 1 & 0_{\mathrm{1\times 12}}\\ 0_{\mathrm{1\times 6}} & 0 & 0 & 1 & 0_{\mathrm{1\times 6}} \end{array}]+[\begin{array}{cc} {\left[\frac{\partial L_{\Psi }^{n}}{\partial \Psi^{n}}\right]\left[\frac{\partial \Psi_{\mathrm{in}}^{n}}{\partial r^{n}}\right]}_{\mathrm{6\times 3}} & 0_{\mathrm{6\times 12}}\\ 0_{\mathrm{1\times 15}}\\ 0_{\mathrm{1\times 15}} \end{array}\right]$, where $z_{k}$ is the measurement vector composed of the differences of the gravity gradient, height, and bearing; and δΓ is the difference between the gravity gradients measured from the gradiometer and the DB, and it is composed of five independent components ($\Gamma_{NN}$, $\Gamma_{NE}$, $\Gamma_{ND}$, $\Gamma_{EE}$, $\Gamma_{ED}$) of the gravity gradient tensor and $\Gamma_{DD}$. The term $\left[\frac{\partial {\text{L}}_{\text{Ψ}}^{\text{n}}}{\partial {\text{Ψ}}^{\text{n}}}\right]\left[\frac{\partial {\text{Ψ}}_{\text{in}}^{\text{n}}}{\partial {\text{cb}}_{\text{u}}}\right]{\text{cδb}}_{\text{u}}$ represents the gravity gradient error caused by the user clock error. ${\text{Ψ}}_{\text{in}}^{\text{n}}$ and ${\text{Ψ}}^{\text{n}}$ are the skew-symmetric rotation error matrix from the navigation-to-inertial frame with coordinates in the n-frame and the navigation-to-body frame rotation error matrix, respectively. c  is the speed of light; ${\text{δb}}_{\text{u}}\;$ is the user clock error; and h and γ denote the height and bearing, respectively. ${\text{H}}_{\text{k}}$ is the design matrix that shows the relation between the measurement and the states. The components of the design matrix are the slope of the gravity gradient in the latitudinal and longitudinal directions ($\frac{\partial {\text{Γ}}_{\text{map}}}{\partial \text{φ}},\;\frac{\partial {\text{Γ}}_{\text{map}}}{\partial \text{λ}})$ and the rotation error term in terms of the three-dimensional (3D) position (r). ${\text{x}}_{\text{k}}$ is the 15-state vector and ${\text{v}}_{\text{k}}$ is the measurement noise vector (assumed to be a white Gaussian process with a zero mean and variance R).

As shown in Equation (2), a primary advantage of GGRN over TRN is the availability of multiple measurements. Besides the measurement vector and the DB, the main idea of both GGRN and TRN is identical. In other words, both systems estimate the state vector with the measurement vector of difference between the observations from the sensors and the DB. Table 1 provides the differences between GGRN and TRN in terms of the DB, the measurement, and the design matrix. In Table 1, $H_{map}$, $H_{baro}$, and $H_{radar}$ are heights from the DB, the barometric altimeter and the radar altimeter, respectively. While TRN uses only one measurement (the difference between the height from both the barometric and the radar altimeters and that from the topographic DB), GGRN uses five or more measurements. Filter-based navigation can diverge or show poor performance when the direction of the slope of the physical data at the INS-indicated position is different from that of the physical data at the true position. Therefore, calculating the correction based on only one measurement may cause an inadequate correction in the filter. From this perspective, the six measurements from the GGI could complement each other. Therefore, GGRN is expected to be theoretically more beneficial and stable than TRN.

**Table 1:** Comparison between GGRN and TRN.

	GGRN	TRN
DB	Gravity Gradient $\left(L_{6\times 1\;}\right)$	Height $\left(H_{map}\right)$
Measurement	$\delta \Gamma =\Gamma_{map,\;6\times 1\;}-L_{6\times 1\;}$	$\delta H=H_{map}-(H_{baro}-H_{radar})$
Design Matirx	$H_{k}=\left[\begin{array}{cc} {\frac{\partial \Gamma_{map}}{\partial \varphi }}_{6\times 1} & {\frac{\partial \Gamma_{map}}{\partial \lambda }}_{6\times 1} \end{array}\right]+\left[\frac{\partial L_{\Psi }^{n}}{\partial \Psi^{n}}\right]\left[\frac{\partial \Psi_{in}^{n}}{\partial r^{n}}\right]$	$H_{k}=\left[\begin{array}{cc} \frac{\partial H_{map}}{\partial \varphi } & \frac{\partial H_{map}}{\partial \lambda } \end{array}\right]$

## 3. Performance Analysis of GGRN

### 3.1. Influencing Factors and Simulation Environments 

To determine the effects of various factors on the navigation performance, the DB and sensor errors, flight altitude, DB resolution, initial errors, and measurement update rate are considered as factors in the simulation. First, the effects of errors in the gravity gradient DB and gradiometer measurement on the navigation results are analyzed. In particular, it is necessary to establish the required accuracies for the DB and sensor to evaluate the performance of GGRN. Therefore, various levels of DB/sensor errors—from 0.1 E to 5 E and from 0.01 E to 5 E, respectively—are considered on the basis of the specifications of the gradiometers that are currently available and under development [10]. Second, the effect of flight altitude is analyzed under the effect of changing gravity field with a change in height. Various flight altitudes of 100 m, 1000 m, 2000 m, and 3000 m are tested in the simulation. Many studies emphasize that an accurate and high-resolution DB is necessary for accurate positioning in DBRN [16,17,18]. Therefore, the effect of the DB resolution is analyzed by employing DBs with resolutions of 9 arcsec, 30 arcsec, and 60 arcsec in the simulation. In general, the performance of GGRN relies strongly on the INS because it uses the position from the INS to extract gravity gradient information from the DB. Specifically, the INS calculates the current position based on a prior position, and so, the performance will degrade when initial position, velocity, and attitude errors exist. In this kind of situation, GGRN is required to compensate for these errors and determine the position of the vehicle accurately. Therefore, the effect of initial errors is analyzed to evaluate the stability of GGRN and its convergence. Various magnitudes of horizontal errors—200 m, 500 m, 900 m, and 1800 m—are applied considering the resolution of both the topography and the gravity gradient DBs. Nine hundred meters is the resolution of the basic gravity gradient DBs, 500 m and 1800 m are the values that are approximately half and double of the gravity gradient DB resolution. Two hundred meters is an approximate value that is twice of the topography DB resolution. In addition, a vertical initial position error of 15 m, horizontal velocity error of 1 m/s, vertical velocity error of 2 m/s, roll and pitch error of 0.1°, and yaw error of 0.5° are applied to evaluate the stability of the GGRN performance. Finally, the effect of the measurement update rate as the fifth factor is analyzed. In the construction of a geophysical DBRN system, the measurement update rate is commonly set to 1 s. However, it may be a better option to update with an intermittent rate if a similar performance could be guaranteed. Therefore, longer measurement update rates—10 s, 20 s, and 50 s—are considered in the simulation tests.

Since several factors or parameters affect the navigation performance, one has to fix other factors to investigate the effects of a specific factor. Therefore, when analyzing the effects of the DB and sensor errors and flight altitude, the DB resolution and measurement update rate are fixed to 30 arcsec and 1 s, respectively. On the other hand, when analyzing the effects of the DB resolution, initial errors, and measurement update rate, the DB and sensor errors are set to the minimum possible values (0.1 E and 0.01 E, respectively). Here, the flight altitude is taken as 3000 m because the main application of GGRN is assumed to be aircraft navigation. As seen in Table 2, a total of 33 combinations are generated and the effects of the factors on the navigation solutions are analyzed. The navigation performance from GGRN with these combinations is compared with that from TRN. Considering the currently available sensor specifications of TRN, the DB and sensor errors are set to 5 m and 10 m, respectively. 

**Table 2:** Test cases based on various factors for simulation test.

Factor	Test Cases	Reference
DB–Sensor error	0.1–0.01, 1–0.1, 3–3, 5–5 [E]	Fix parameters • DB resolution: 30 (arcsec) • Measurement update rate: 1 (s)
Flight altitude	100, 1000, 2000, 3000 (m)	
DB resolution	9, 30, 60 (arcsec)	Fix parameters • DB-Sensor error : 0.1–0.01 [E] • flight altitude : 3000 (m)
Initial errors	• Position - Horizontal : 200, 500, 900, 1800 (m) - Vertical : 15 (m) • Velocity - Horizontal : 1 (m/s) - Vertical : 2 (m/s) • Attitude - Roll, Pitch : 0.1 (°) - Yaw : 0.5 (°)	
Measurement update rate	1, 10, 20, 50 (s)	

In the simulation, the moving vehicle is assumed to be an aircraft equipped with a navigation-grade INS, FTG, altimeter sensor, and compass sensor. The flight trajectory is assumed to be a straight line, and the speed of the flight is set to 350 km/h. The errors of the altimeter and compass are assumed to be 5 m and 0.5 °/h, respectively. The specifications of the sensors assumed in this study are shown in Table 3 and Table 4.

**Table 3:** Specifications of the INS assumed in the simulation.

Sampling Rate (Hz)	Accelerometer		Gyro	
	Bias (mg)	VRW (m/s/$\sqrt{hr}$)	Bias (°/h)	ARW (°/$\sqrt{hr}$)
50	0.0022	0.005	0.003	0.0015

**Table 4:** Specifications of the geophysical sensors assumed in the simulation.

Sensor Type	Sampling Rate (Hz)	Accuracy
Gravity gradiometer	1	0.01, 0.1, 3, 5 [E]
Barometric altimeter	1	5 (m)
Compass	1	0.5 (°)

**Figure 2:**
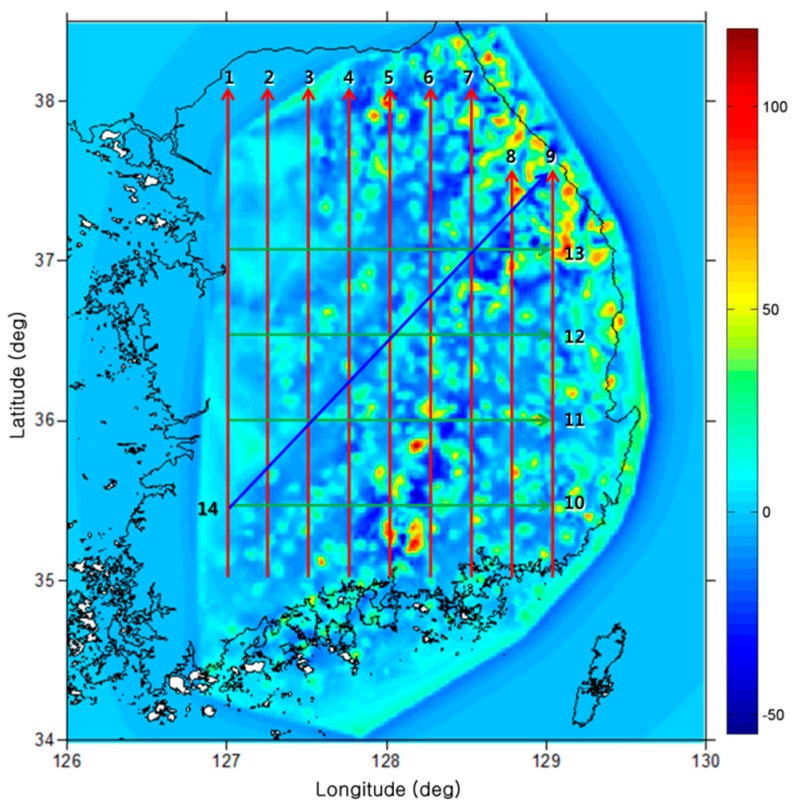
Distribution of trajectories and gravity gradient ($\Gamma_{DD}$, unit: E).

Figure 2 shows the gradient of the down component of the gravity vector in down direction, $\Gamma_{DD}$, at an altitude of 3000 m in the test area with the flight trajectories. As the variations of the gravity gradient would have an effect on the navigation results, a total of 14 trajectories are generated for the tests. Out of these 14 trajectories, nine that fly from south to north are distributed with a 0.25° interval from longitude 127°. Among these nine trajectories, seven trajectories are from latitudes 35° to 38°, and the remaining two are from latitudes 35° to 37.5° to avoid the ocean area. Most trajectories show relatively small variations of the gravity gradients at the starting points, but large local variations occur as the flight passes mountainous areas. Out of the 14 trajectories, four are designed from west to east with latitudes from 127° to 129°; the latitudes of these trajectories are 35.5°, 36°, 36.5°, and 37°, respectively. The final of the 14 trajectories is generated to fly from southwest to northeast. Because the gravity gradient increases going toward the northeastern area, trajectory 14 experiences a huge variation in the gravity gradient from the second half of the flight duration.

The performance of GGRN is evaluated on the basis of 2D position errors with respect to the simulated true trajectories. The standard deviation of the horizontal errors for each trajectory is calculated using the following Equation (3):

(3) $$\text{Δ}\sigma_{hor}=\sqrt{\frac{\sum_{1}^{n}{\left(\text{Δ}d-\hat{\text{Δ}d}\right)}^{2}}{n-1}}$$

where Δd is the horizontal position error calculated by the sum of differences between the horizontal position calculated by GGRN and the true horizontal position in the latitude and longitude, $\hat{\Delta d}$ is the mean of the horizontal position error, and n is the total number of epochs.

### 3.2. Performance Analysis of GGRN

From the simulation results, it is found that accurate positioning is possible with small DB and sensor errors and a lower flight altitude. In the case of an accurate DB and sensor with errors of 0.1 E and 0.01 E, respectively, all the trajectories show horizontal errors of less than 10 m irrespective of the flight altitude. However, the navigation results degrade when the flight altitude increases and the DB and sensor errors increase. For example, the navigation performance remains at the level of several hundred meters when the DB and sensor errors are set to 3 E and 5 E, respectively, and the flight altitude is 3000 m. 

As mentioned previously, the navigation results are also generated using TRN for comparison purposes, and the results from TRN show a horizontal accuracy of 10–20 m. Comparison of the results from GGRN and TRN shows that the GGRN exhibits better performance when the flight altitude is 100 m. In addition, GGRN and TRN exhibit similar levels of performance at altitudes higher than 1000 m only when a high-accuracy DB (0.1 E) and gradiometer (0.01 E) are used. This means that GGRN with currently available sensors and DBs is not comparable to TRN in terms of navigation performance. However, GGRN could perform better than TRN post the development of a gravity gradiometer with an accuracy better than 0.01 E and construction of a DB with an accuracy better than 0.1 E in the future. 

**Figure 3:**
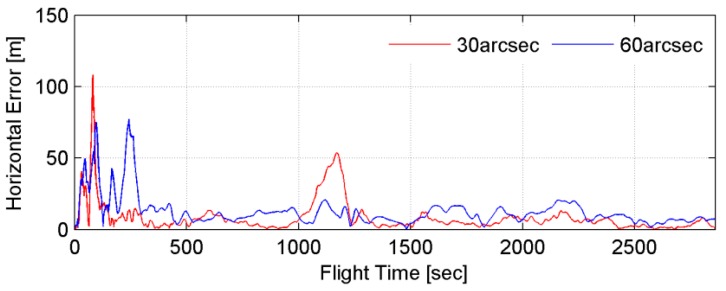
Horizontal error as a function of DB resolution in trajectory No. 9.

In general, better navigation results are expected when the DB resolution is higher. However, navigation performance in the case of using low-resolution DBs is sometimes better when the data changes abruptly. Figure 3 shows the horizontal position error of trajectory No. 9. In this figure, the red and blue lines indicate the horizontal errors corresponding to the application of 30 arcsec and 60 arcsec DBs, respectively. As shown in the figure, the positions of the vehicle are determined more precisely with a higher-resolution DB for a majority of the navigation time. However, the horizontal error for the 60-arcsec-resolution DB is smaller in the time window between 1000 s and 1300 s. This kind of event is commonly observed in situations where the linearity between the measurements and the states is broken. In other words, the true aircraft position and the INS-indicated position could be located on the same slope in a lower-resolution DB but they could be located on opposite slopes in a higher-resolution DB. Therefore, it would be a better to use a low-resolution DB in the abruptly changing region to obtain stable navigation results. 

**Figure 4:**
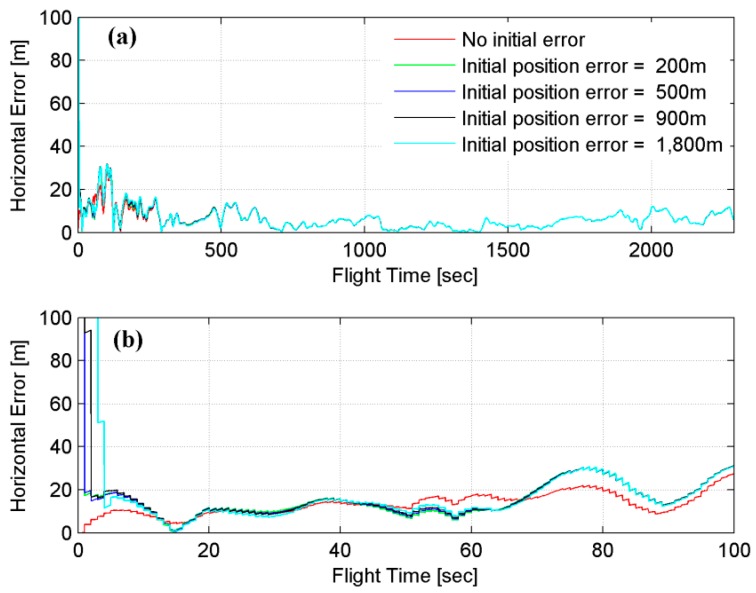
Horizontal error of trajectory No. 12: (**a**) entire flight and (**b**) before 100 s.

As expected, better navigation results are obtained when initial errors are not imposed. However, the initial errors are compensated for rapidly in the case of DB and sensor errors of 0.1 E and 0.01 E, respectively. Figure 4 shows the horizontal error of trajectory No. 12 when four different initial position errors are imposed. It is found that the effect of the initial error appears mostly in the starting zone, and the performance improves once the filter converges; therefore, similar navigation results are obtained despite a large initial horizontal position error of 1800 m. When the worst possible accuracies are considered for the sensor and DB, the positions frequently diverge in the case of imposing initial errors. Therefore, it is crucial to develop and construct an accurate sensor and DB to correct the initial errors.

Most trajectories showed poorer navigation performance when the measurement update rates became longer. However, some of them provided better navigation results when longer measurement update rates were applied. Therefore, it would be better to determine a specific update time instead of applying fixed measurement update rates at all times. It should be noted that a frequent update is required to compensate for an error rapidly if initial errors exist (see the case of horizontal initial position error of 200 m in Table 6). More detailed analysis of the effect of each influencing factor can be found in [15,19].

## 4. Assessment of Requirements for GGRN

As revealed in the previous section, various factors have a complex effect on the navigation performance. In this study, the above-presented simulation results are reanalyzed from a viewpoint of establishing the requirements for achieving a certain target accuracy in GGRN. The target accuracy is divided into five groups, and the condition of each group is set as given in Table 3. For example, Group 1 is the one that includes better navigation results than those obtained from TRN. Because the accuracy of TRN is known to be on the order of several tens of meters, the criteria for the mean and maximum horizontal errors for Group 1 are set to 5 m and 10 m, respectively. For Group 2, the mean horizontal error is set in the range of 5–10 m and the maximum horizontal error is set to 50 m. Group 2 includes results that show a similar level of accuracy to that of results from TRN. Group 3, which generates a stable navigation performance, shows a horizontal error of less than 50 m. Furthermore, it is required that 80% of trajectories in Group 3 should have horizontal error within 100 m. In particular, a condition of the mean horizontal error being more than 50 m is defined for Group 3 when entire trajectories determine the position with a horizontal error of less than 100 m. In Group 4, the mean horizontal error is set as being over 50 m, but this group should show better performance than pure INS navigation. Finally, Group 5 includes poor navigation results, because of which divergence appears in some trajectories.

Table 6 presents the requirements of flying conditions for achieving the target accuracies categorized in Table 5. When the flight altitude is 100 m, GGRN shows better performance than TRN if the DB and sensor errors are smaller than 1 E and 0.1 E, respectively. In particular, entire trajectories show a horizontal error of less than 1 m when the DB and sensor errors are set to the minimum possible values, *i.e.*, 0.1 E and 0.01 E, respectively. If the flight altitude increases, however, a more accurate DB and a sensor having error levels of 0.1 E and 0.01 E, respectively, are required. In addition, a high-resolution DB is necessary for a flight altitude of 3000 m. It should be mentioned that the fundamental requirements for generating the navigation results categorized into Group 1 are the accurate DB and sensor that are expected to be available in the future. 

**Table 5:** Description of each target accuracy group.

Group	Definition	Conditions
1	More accurate than TRN	Mean horizontal position error is less than 5 m, maximum error is less than 10 m
2	Similar accuracy to TRN	Mean horizontal position error is less than 5–10 m, maximum error is less than 15 m
3	Stable performance	Mean horizontal position error is 10–50 m, 80% of trajectories show less than 100 m of horizontal position error (Exception: mean horizontal position error is over 50 m but whole trajectories show less than 100 m)
4	Limit of GGRN	Mean horizontal position error is over 50 m, no divergence
5	Non-applicable	Number of divergence trajectories are more than five

When the target accuracy ranges from 5 m to 10 m, the currently developed GGI, which has an accuracy of 3 E or 5 E, will be applicable for GGRN only for the flight altitude of 100 m. However, the DB and sensor errors should be limited to 1 E and 0.1 E, respectively, at 1000 m, and to 0.1 E and 0.01 E, respectively, at 3000 m, because the variation of the gravity gradient becomes smoother with an increase in the flight altitude. When the flight altitude is 3000 m, it would be possible to obtain accurate results that do not exceed 50 m with a measurement update rate of 20 s, as long as the DB and sensor errors are 0.1 E and 0.01 E, respectively. This means that high computational efficiency could be achieved when the DB and sensor are sufficiently accurate. 

It should be noted that most test combinations are included in Group 3. In other words, it would be possible to obtain a stable navigation performance with GGRN. When the flight altitude is 1000 m, a commercial gradiometer can be employed for the navigation. However, a more accurate DB and sensor are still required at the altitude of 2000 m, and so, the DB and sensor errors should be smaller than 3 E. When the flight altitude is 3000 m, a DB and sensor having accuracies of 1 E and 0.1 E, respectively, would be applicable to GGRN. However, a high DB resolution of 30 arcsec and every epoch update are both necessary. On the other hand, the DB and sensor errors should be limited to the minimum possible values when a longer update (up to 50 s) and lower-resolution DB at the same flight altitude are assumed. Furthermore, a high-accuracy DB and gradiometer are required when an initial horizontal position error exists. This implies that the effect of factors such as the measurement update rate, DB resolution, and initial error could be compensated for with a high-accuracy DB and sensor. For example, if the DB and sensor are sufficiently accurate, it is possible to obtain results belonging to Group 3 even when the initial horizontal position error is 900 m. 

**Table 6:** Requirements for GGRN to achieve target accuracy of each group.

Group	Altitude (m)	Initial Error	DB-Sensor Error [E]	DB Resolution (arcsec)	Measurement Update Rate (s)	Horizontal Error (m)	
						Max	Mean
1	100	None	0.1–0.01	30	1	0.91	0.39
			1–0.1	30	1	6.64	2.62
	1000	None	0.1–0.01	30	1	3.60	1.56
	2000	None	0.1–0.01	30	1	9.19	4.56
	3000	None	0.1–0.01	9	1	7.86	4.29
2	100	None	3–3	30	1	21.89	6.41
			5–5	30	1	36.40	9.61
	1000	None	1–0.1	30	1	24.32	9.52
	3000	None	0.1–0.01	30	1	15.28	8.44
				30	10	13.17	7.09
				30	20	27.18	9.75
3	1000	None	3–3	30	1	86.42	23.89
			5–5	30	1	130.62	33.65
	2000	None	1–0.1	30	1	93.55	26.89
			3–3	30	1	136.11	48.08
	3000	None	0.1–0.01	30	50	48.85	16.28
			0.1–0.01	60	1	45.08	14.99
			1–0.1	30	1	141.92	46.50
		Horizontal 200 m	0.1–0.01	30	1	22.22	11.23
				30	10	32.51	21.21
				30	20	56.05	30.60
				30	50	91.69	51.17
				9	1	49.14	12.25
				60	1	49.32	17.02
		Horizontal 500 m	0.1–0.01	30	1	28.22	17.66
		Horizontal 900 m	0.1–0.01	30	1	49.29	28.33
4	2000	None	5–5	30	1	152.02	79.51
	3000	None	3 3	30	1	192.68	106.77
			5–5	30	1	865.31	294.35
		Horizontal 200 m	1–0.1	30	1	193.35	62.61
			3–3	30	1	537.62	285.04
		Horizontal 1800 m	0.1–0.01	30	1	233.31	68.24
5	3000	Horizontal 200 m	5–5	30	1	3872.08	1178.95

If the DB and sensor errors are at the current accuracy and the flight altitude is higher than 2000 m, GGRN generates similar results to pure INS navigation. In the case that the DB and sensor errors are 5 E each and the initial horizontal error is 200 m, the filter diverges frequently. Therefore, applicability of GGRN should be considered with an accuracy of 3 E or higher. Again, it should be mentioned that it is important to determine the initial position, velocity, and attitude of the aircraft if the DB and sensor have insufficient accuracy. 

To sum up, the most important factors in GGRN are the accuracies of the DB and sensor and the flight altitude. Because the gravity signal becomes smoother with an increase in flight altitude, it would be better to fly at a lower altitude when a currently available sensor is applied to the navigation. On the other hand, the DB and sensor errors should be better than 1 E and 0.1 E for navigation above an altitude of 1000 m. When the sensor and DB are sufficiently accurate, it is possible to apply longer measurement update rates for achieving high computational efficiency. 

## 5. Conclusions

To evaluate the performance of GGRN, various tests are conducted in which the DB and sensor errors, flight altitude, DB resolution, initial errors, and measurement update rates are considered as influencing factors. The simulation results show that better performance is achieved when the DB and sensor errors are smaller and the flight altitude is lower. In particular, navigation results similar to or better than those from TRN are obtained when DB and sensor errors of 0.1 E and 0.01 E, respectively, are applied. Although the performance is generally better when a high-resolution DB is used, it would be better to use a low-resolution DB when the gravity gradient shows sudden irregular variation. If the DB and sensor are sufficiently accurate, GGRN generates accurate results even in the presence of initial errors. It is better to apply measurement update every epoch, especially in the presence of initial errors. The simulation results are divided into groups to establish conditions that meet the requirement of achieving certain target accuracies. When the DB and sensor accuracies are 0.1 E and 0.01 E, respectively, GGRN shows better performance than TRN. With the currently available DB and sensor accuracies, GGRN shows comparable performance to TRN only at the flight altitude of 100 m. If the flight altitude is lower than 2000 m, the currently available GGI would generate a horizontal position error of about 100 m that would be applicable to the navigation. This means that GGRN would be applied to a low flying airplane, an unmanned aerial vehicle, and a submarine with current technology, especially in non-GNSS environment. It is found that the currently available sensor cannot be implemented in GGRN at altitudes of 3000 m or higher. Although GGRN with the current technology is not comparable to other forms of database referenced navigation, such as TRN, it would still be beneficial to utilize gravity information for achieving more stable navigation. Therefore, a study on database referenced navigation based on a combination of terrain and gravity gradients is proposed as future research.
